# Investigating Language and Domain-General Processing in Neurotypicals and Individuals With Aphasia — A Functional Near-Infrared Spectroscopy Pilot Study

**DOI:** 10.3389/fnhum.2021.728151

**Published:** 2021-09-17

**Authors:** Natalie Gilmore, Meryem Ayse Yücel, Xinge Li, David A. Boas, Swathi Kiran

**Affiliations:** ^1^Department of Speech Language & Hearing Sciences, Sargent College of Health and Rehabilitation Sciences, Boston University, Boston, MA, United States; ^2^Neurophotonics Center, Biomedical Engineering, Boston University, Boston, MA, United States; ^3^Department of Psychology, College of Liberal Arts and Social Sciences, University of Houston, Houston, TX, United States

**Keywords:** near infrared-spectroscopy, language, cognition, healthy subjects, stroke, aphasia

## Abstract

Brain reorganization patterns associated with language recovery after stroke have long been debated. Studying mechanisms of spontaneous and treatment-induced language recovery in post-stroke aphasia requires a network-based approach given the potential for recruitment of perilesional left hemisphere language regions, homologous right hemisphere language regions, and/or spared bilateral domain-general regions. Recent hardware, software, and methodological advances in functional near-infrared spectroscopy (fNIRS) make it well-suited to examine this question. fNIRS is cost-effective with minimal contraindications, making it a robust option to monitor treatment-related brain activation changes over time. Establishing clear activation patterns in neurotypical adults during language and domain-general cognitive processes via fNIRS is an important first step. Some fNIRS studies have investigated key language processes in healthy adults, yet findings are challenging to interpret in the context of methodological limitations. This pilot study used fNIRS to capture brain activation during language and domain-general processing in neurotypicals and individuals with aphasia. These findings will serve as a reference when interpreting treatment-related changes in brain activation patterns in post-stroke aphasia in the future. Twenty-four young healthy controls, seventeen older healthy controls, and six individuals with left hemisphere stroke-induced aphasia completed two language tasks (i.e., semantic feature, picture naming) and one domain-general cognitive task (i.e., arithmetic) twice during fNIRS. The probe covered bilateral frontal, parietal, and temporal lobes and included short-separation detectors for scalp signal nuisance regression. Younger and older healthy controls activated core language regions during semantic feature processing (e.g., left inferior frontal gyrus pars opercularis) and lexical retrieval (e.g., left inferior frontal gyrus pars triangularis) and domain-general regions (e.g., bilateral middle frontal gyri) during hard versus easy arithmetic as expected. Consistent with theories of post-stroke language recovery, individuals with aphasia activated areas outside the traditional networks: left superior frontal gyrus and left supramarginal gyrus during semantic feature judgment; left superior frontal gyrus and right precentral gyrus during picture naming; and left inferior frontal gyrus pars opercularis during arithmetic processing. The preliminary findings in the stroke group highlight the utility of using fNIRS to study language and domain-general processing in aphasia.

## Introduction

Cognitive processes, including language, are supported by large-scale brain networks (Mesulam, 1990; Greicius et al., 2003; Fox et al., 2005; Yeo et al., 2011). While the language network can be challenging to clearly define (Yeo et al., 2011), a subset of regions seems to consistently engage during language production and expression activities (i.e., left inferior frontal; IFG and middle temporal gyri; MTG; Fedorenko and Thompson-Schill, 2014; Ji et al., 2019). Depending on the demands of the activity, the language network expands to include areas important for sensory/motor functions, domain-general cognitive control, and even social cognition (Mesulam, 1990; Fedorenko and Thompson-Schill, 2014; Braga et al., 2020). For example, and relevant to the present study, lexical retrieval and semantic processing in healthy individuals includes not only LIFG and MTG, but also, middle frontal (MFG), precentral (PCG), supramarginal (SMG), and angular gyri (AG; see Johnson et al., 2019, 2020). Furthermore, language activities may be supported by the domain-general cognitive control network (MFG, inferior frontal gyrus opercularis [IFGoper], PCG, supplementary motor area [SMA], insula, superior parietal lobule, SMG, AG, anterior cingulate cortex [ACC]; Fedorenko et al., 2013) as this bilaterally represented network is engaged when tasks are challenging irrespective of modality. Fedorenko et al. (2011, 2013) have demonstrated this phenomenon extensively across a range of cognitive domains (e.g., spatial working memory, verbal working memory, Stroop), including arithmetic as investigated in the present study.

When large-scale brain networks are damaged, as in the context of stroke, disruptions in white matter connections can lead to impaired function (Siegel et al., 2016). In the case of the language network, left-hemisphere stroke may result in aphasia, or deficits in linguistic processes (Mirman and Thye, 2018), such as phonology, semantics, and syntax, that subserve language domains like verbal expression, auditory comprehension, reading comprehension, and written expression. Language recovery in post-stroke aphasia is supported by neuroplasticity, or the brain’s ability to adapt and reorganize in response to experience (Kiran and Thompson, 2019). Based on a detailed review of the neuroimaging literature in aphasia, Kiran et al. (2019) suggest that aphasia recovery may include recruitment of (1) perilesional left hemisphere language regions (e.g., MTG; Kiran et al., 2015); (2) homologous right hemisphere language regions (e.g., IFGoper; Turkeltaub et al., 2011); and/or (3) spared non-language left hemisphere regions (e.g., superior frontal gyrus [SFG]; Szaflarski et al., 2013) or bilateral domain-general regions (e.g., ACC; Geranmayeh et al., 2017).

The bulk of neuroimaging studies investigating patterns of reorganization associated with language recovery in aphasia have used task-based functional magnetic resonance imaging (fMRI; Wilson and Schneck, 2020). fMRI has clearly been valuable for studying neuroplasticity in this population. Yet, several disadvantages may limit the individuals and experimental conditions that can be investigated with this neuroimaging modality (Irani et al., 2007). For example, it exposes individuals to loud noises, which can be detrimental to task performance for individuals with hearing impairment and/or auditory comprehension deficits post-stroke. It also requires individuals to lie still and flat, which can be difficult in the context of post-stroke pain, paralysis, and/or cognitive deficits, and lead to motion artifacts. Further, individuals with ferromagnetic material (e.g., surgical clips) and/or electronic medical implants (e.g., pacemaker) may be excluded from fMRI studies due to safety concerns (Carr and Grey, 2002), which could impact study recruitment and generalizability of study results.

Functional near-infrared spectroscopy (fNIRS) is an alternative to fMRI that relies on the optical properties of hemoglobin to monitor neural activity (Boas et al., 2014). Near-infrared light at multiple wavelengths^1^ diffuses through the scalp into the cortex. The remaining portion of light not absorbed by tissue is captured by detector optodes placed on the scalp. One can then estimate the concentration changes in oxygenated (HbO), deoxygenated (HbR), and total hemoglobin (HbT) using the modified Beer-Lambert Law (Cope and Delpy, 1988; Delpy, 1988; Boas et al., 2004). fNIRS is ideally suited to study language and other cognitive processing, especially in clinical populations like post-stroke aphasia (Arenth et al., 2007; Irani et al., 2007). It is quiet, can be performed in a natural environment, and is safe in the context of metal or other implanted material. While not without limitation (e.g., cannot measure deeper structures, has poorer spatial resolution than fMRI), fNIRS hardware continues to rapidly evolve to address these challenges (Pifferi et al., 2016; Ladouce et al., 2017; Yücel et al., 2017; Zhao and Cooper, 2017; Pinti et al., 2018; von Lühmann et al., 2021).

Numerous studies have used fNIRS to investigate what brain areas are contributing to various language (e.g., lexical retrieval, semantic processing) and domain-general cognitive control processes (e.g., arithmetic complexity) in neurotypical adults (Dieler et al., 2012; Quaresima et al., 2012; Rossi et al., 2012; Pinti et al., 2020). Supplementary Table 1 reviews studies that assessed brain activation during lexical retrieval, semantic processing, and hard versus easy arithmetic (i.e., domain-general cognitive processing during complex task completion)— the three constructs of interest in the present study. Lexical retrieval fNIRS studies in neurotypicals have demonstrated engagement of left-lateralized frontal and temporal areas during picture naming in general agreement with previous work in this area using fMRI and other neuroimaging modalities. However, additional investigation of lexical retrieval in neurotypicals is necessary as previous fNIRS studies in this area have (1) predominantly focused on a narrow aspect of the language network (e.g., LIFG); (2) typically compared hemodynamic response function (HRF) in the experimental condition to rest/baseline as opposed to an active control condition; and/or (3) not employed techniques to separate spurious scalp signal from the cortical signal (e.g., application of short-separation channel regression; Yücel et al., 2015). Only a few studies have investigated semantic processing in healthy adults using fNIRS (Kennan et al., 2002; Noguchi et al., 2002) and not all have reported robust results (Noguchi et al., 2002). Thus, more work examining the semantic system in neurotypicals via fNIRS is necessary to serve as a reference when interpreting activation patterns in impaired populations. Finally, inherent to its name, the domain-general network may be recruited for a wide range of mental activities and fNIRS has been used to investigate many of them (Himichi et al., 2015; Noah et al., 2017; Li et al., 2019; Stute et al., 2020). Relevant to the present study, two studies investigating the neural bases of arithmetic complexity in neurotypical adults (i.e., hard versus easy addition/subtraction; Artemenko et al., 2018; hard versus easy multiplication/division; Artemenko et al., 2019 found that arithmetic processing recruits bilateral parietal areas (i.e., SMG, AG) at a base level and inferior frontal regions (i.e., LIFG) when task complexity increases. These studies were primarily focused on how brain activation patterns associated with task difficulty differed as a function of math ability in young healthy controls, and thus, findings from the present study, investigating different age groups and individuals with stroke-induced aphasia, will complement this work.

Beyond the pragmatic benefits previously mentioned, fNIRS also provides scientific advantages to the study of neural activity. For example, it provides a more thorough evaluation of the hemodynamic response than fMRI with measurement of both oxygenated and deoxygenated hemoglobin concentration change [i.e., blood-oxygen dependent (BOLD) signal in fMRI relies on deoxygenated hemoglobin only; Quaresima and Ferrari, 2019]. According to recently published reviews (Pinti et al., 2018; Quaresima and Ferrari, 2019), fNIRS also has better temporal resolution (i.e., sampling rate of 1–10 Hz with maximum of 100 Hz; 50 Hz in this study) than fMRI [i.e., sampling rate of 1–3 Hz; 0.5 Hz in Kiran et al. (2015) which used two of the tasks employed in the present study]. This oversampling in fNIRS allows for (1) the separation of the evoked signal from physiological noise and motion artifacts and (2) the observation of the onset and shape of the HRF (Wilcox and Biondi, 2015; Pinti et al., 2018). Overall, these advantages well-position fNIRS to capture the potentially altered hemodynamic response in the post-stroke population (D’Esposito et al., 2003; Bonakdarpour et al., 2007; Obrig and Steinbrink, 2011; Yang et al., 2019).

Despite these advantages, only two published fNIRS studies have included individuals with post-stroke aphasia (Sakatani et al., 1998; Hara et al., 2017) and neither of them investigated semantic or domain-general cognitive processing as in the present study (see Dieler et al., 2012; Yang et al., 2019; Butler et al., 2020 for relevant reviews). In a seminal paper investigating language processing in individuals with aphasia using fNIRS, Sakatani et al. (1998) found that post-stroke individuals with aphasia (*n* = 10) exhibited significantly higher deoxygenated hemoglobin concentration change than healthy participants (*n* = 13) and post-stroke individuals without aphasia (*n* = 6) in left prefrontal cortex during confrontation naming. According to the authors, this finding suggests that the left prefrontal cortex was more active during naming in the post-stroke aphasia group than in the other two groups. Further, there were no significant differences in oxygenated hemoglobin concentration change between the groups in this region during naming, highlighting the benefit of measuring both chromophores (i.e., HbO and HbR). Hara et al. (2017) expanded the use of fNIRS in the study of aphasia (*n* = 8) by applying it to localize language activation in superior temporal gyrus and IFG during a word repetition task with half of the chronic stroke participants showing activation in the left hemisphere and the other half showing activation in the right hemisphere. While these studies suffered some methodological limitations [e.g., Sakatani et al. (1998) predominantly analyzed activation patterns qualitatively; the Hara group did not discuss management of lesion in the fNIRS data analysis], they provided preliminary support for the application of fNIRS to assess overt language production in individuals with post-stroke aphasia as was conducted in the present study.

In sum, this neuroimaging modality has the potential to significantly advance the field of aphasia rehabilitation. However, before it can begin to be applied extensively to study recovery and inform rehabilitation paradigms in post-stroke aphasia, it is important to establish a firm understanding of the activation patterns exhibited by healthy adults during language and domain-general processing as captured by fNIRS. In response, this study applied fNIRS to capture brain activation in expected regions in neurotypicals and post-stroke individuals during language and domain-general cognitive tasks. It was also intended to address methodological limitations of previous work in this area by implementing a novel method to manage lesioned areas of the brain during fNIRS data analysis as detailed in section “Management of the lesion” of the Methods.

First, this study investigated what areas of the brain were recruited for language and domain-general processing by healthy individuals as measured by fNIRS and whether these activation patterns were similar to those identified in previous neuroimaging studies using these tasks. Young healthy controls were anticipated to activate a network of regions spanning the frontal, parietal, and temporal lobes during semantic and lexical retrieval processing (Indefrey and Levelt, 2004; Binder et al., 2009; Price, 2012). When performing hard versus easy addition, young healthy controls were expected to activate the domain-general network (i.e., bilateral MFG, IFGoper, PCG, SMG, and AG; Fedorenko et al., 2011, 2013). Similar to younger healthy controls, older healthy controls were hypothesized to activate core language areas (i.e., left SFG, IFGtri, PCG; bilateral MFG, IFGoper, MTG, SMG, and AG; Sebastian and Kiran, 2011; Kiran et al., 2015; Meier et al., 2018, 2019) with potential for some right hemisphere (Hoffman and Morcom, 2018) and bilateral engagement (Velanova et al., 2007; Spreng et al., 2010) not seen in the younger healthy controls.^2^

Finally, the study investigated what areas of the brain were recruited for language and domain-general processing by individuals with aphasia as measured by fNIRS. While similar cortical findings to those identified in previous fMRI studies were expected, it was hypothesized that fNIRS’ capacity to interrogate both oxygenated and deoxygenated hemoglobin coupled with its superior temporal resolution may yield novel information about the hemodynamic response during language and domain-general cognitive processing in individuals with post-stroke aphasia. In the language tasks, several different activation patterns were expected. Extrapolated from fMRI studies, individuals with aphasia were hypothesized to recruit spared left hemisphere language regions (i.e., LIFGtri, LIFGoper, LPCG, LMTG, LAG, Sebastian and Kiran, 2011; Sims et al., 2016; Kiran et al., 2019; Wilson and Schneck, 2020), right hemisphere homologous language regions (i.e., RMTG; Sebastian and Kiran, 2011) and/or domain-general areas (i.e., LMFG, LIFGoper, BLPCG, LSMG, LAG; Meier et al., 2016, 2018, 2019) while naming or judging semantic features of real pictures. In the arithmetic task, they were anticipated to activate regions in the spared domain-general network when solving hard versus easy addition problems with potential for diminished extent or magnitude of activation relative to healthy controls (Blank et al., 2015). This hypothesis for activation of the domain-general network in individuals with aphasia was supported by the fact that language was their primary impaired cognitive domain and thus, they were not expected to show the same low accuracy in the hard condition of the domain-general task as in the naming and semantic feature judgment conditions of the language tasks, although given the neuroanatomical overlap between the domain-general and language network (e.g., AG), some decrement in task performance relative to controls was possible.

## Materials and Methods

### Participants

Twenty-four young healthy individuals (12M; Mean Age [SD]: 24.7 [4.7], Range: 19–37), seventeen older healthy individuals (7M; Mean Age [SD]: 66.0 [5.8]; Range: 56–74), and six individuals with chronic left-hemisphere stroke-induced aphasia (6M; Mean Age [SD]: 50.8 [21.6]; Mean Months Post Onset [SD]: 131.5 [71.3]) were recruited from the greater Boston area for this study.^3^ Participants in the stroke group were diagnosed with aphasia using the Western Aphasia Battery-Revised Aphasia Quotient (WAB-AQ Mean [SD]: 67.17 [19.48]) with quotients less than 93.8 suggesting the presence of aphasia.^4^ See Table 1 for WAB-AQs for individual participants in the stroke group. All study participants were consented according to a human participants protocol approved by the Boston University Institutional Review Board.

**TABLE 1:** Demographics for individuals with stroke-induced aphasia.

ID	Age (in years)	Time post onset (in years)	WAB-AQ
PWA 1	72.43	17.05	44.60
PWA 2	60.49	16.72	42.80
PWA 3	55.50	14.94	63.60
PWA 4	27.35	7.75	69.80
PWA 5	20.95	4.99	93.20
PWA 6	68.02	4.29	89.00
Mean (SD)	50.79 (21.55)	10.96 (5.94)	67.17 (21.34)
Range	20.95 – 72.43	4.29 – 17.05	42.20 – 93.20

*PWA, persons with aphasia; WAB-AQ, Western Aphasia Battery Aphasia Quotient < 93.8 indicates presence of aphasia.*

### Behavioral Data Acquisition

**FIGURE 1:**
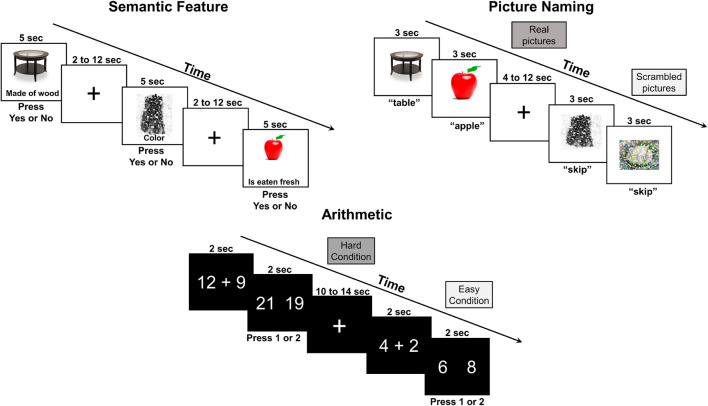
Functional near-infrared spectroscopy behavioral tasks.

During fNIRS measurement, participants completed two runs of a semantic feature judgment, picture naming, and arithmetic task in consecutive order (see Figure 1). All three tasks started and ended with a 15-s interval of rest.

The semantic task employed an event-related design with jittered inter-stimulus intervals ranging from 2 to 12 s (i.e., average ISI of 7 s) and a stimulus duration of 5 s for an overall task time of ∼6 min/run. Each run included 15 real pictures (e.g., sock, fig, spinach) and 15 scrambled pictures (i.e., pixelated images) that were randomly ordered. In the real picture condition, participants were shown a real picture (e.g., apple) with a written semantic feature (e.g., has seeds) and instructed to press ‘yes’ if the semantic feature applied and ‘no’ if it did not. In the scrambled picture condition, participants were shown a color or black/white pixelated image with a written phrase (i.e., “is color” or “is black and white”). Accuracy and reaction time were recorded and scored automatically through E-Prime.

The picture naming task used a block design with 4- to 12-s jittered inter-block intervals and a total block duration of 15 s for an overall task time of ∼5.5–6 min/run. Each run included five trials per block with seven blocks per condition (i.e., real pictures, scrambled pictures). During the real picture blocks, participants were shown real pictures (e.g., table) and asked to name them aloud. During the scrambled picture blocks, they were shown a pixelated image and asked to say “skip” aloud in response. Responses during the picture naming task were recorded using Audacity and scored offline for accuracy. No reaction time data was recorded for the picture naming as it was not of primary interest in this study. See section “fNIRS Data Analysis” fNIRS Data Analysis for additional details regarding motion correction, especially relevant for this task involving overt speech production.

The arithmetic task utilized a block design with 10- to 14-s jittered inter-block intervals and a total block duration of 32 s for an overall task time of ∼6 min/run. Each run included eight trials per block with four blocks per condition (i.e., hard addition, easy addition). In the first frame, participants were given a math problem to solve, and then, in the second frame, they were asked to select the answer by pressing ‘1’ if the correct response was on the left or pressing ‘2’ if it was on the right. The hard condition included two-digit plus single-digit addition problems (e.g., 17 + 7), while the easy condition included single-digit addition problems (e.g., 2 + 4). Accuracy and reaction time were recorded and scored automatically through PsychToolbox-3 via MATLAB.

### Behavioral Data Analysis

Mean accuracy and reaction time were averaged across runs for each participant.^5^ Paired sample *t*-tests were then performed to assess for statistically significant differences in behavioral performance between task conditions for each group. *P*-values were adjusted for multiple comparisons using the Benjamini–Hochberg procedure with a false discovery rate (FDR) of 0.05.

### fNIRS Data Acquisition

**FIGURE 2:**
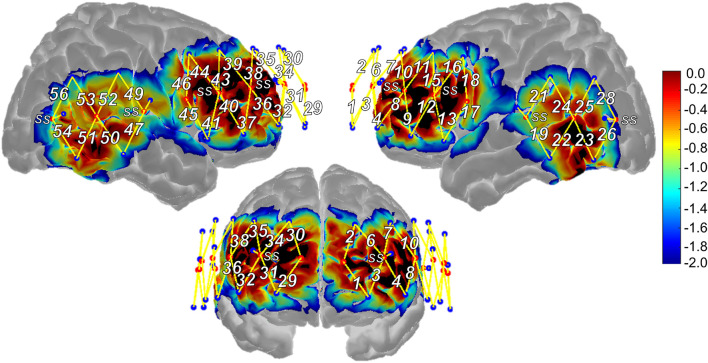
Functional near-infrared spectroscopy probe design and sensitivity profile. SS, short-separation regression channel, Warmer colors suggest higher sensitivity to cortex, cooler colors suggest lower sensitivity to cortex.

fNIRS measurements were acquired using a TechEn continuous-wave NIRS device (TechEn Inc., MA, United States) with a 50 Hz sampling frequency. As shown in Figure 2, the 56-channel probe (i.e., source-detector pairs) covered the frontal, temporal, and parietal lobes bilaterally. Sixteen sources emitted light through participants’ scalp to cortex at 690 and 830 nm wavelengths. Twenty-four long-separation detectors (∼30 mm distance from the source) captured signal from participants’ scalp and cortex. Eight short-separation detectors (∼8 mm distance from the source), split evenly across anterior and posterior aspects of the probe, captured signal from participants’ scalp only. To support optode-to-scalp coupling, sources and detectors were stabilized in a cap (EASYCap GmbH, Woerthsee-Etterschlag, Germany) that participants wore during the measurement.

Following fNIRS measurement, 3D locations of nasion, inion, left/right pre-auricular points, and Cz as well as the sources, long-separation detectors, and short-separation detectors were obtained for each participant using a Polhemus 3-D digitizer (Polhemus, VT, United States) to support subsequent anatomically based interpretation of the fNIRS results. The five landmarks were registered to the Colin brain atlas (Collins et al., 1998), allowing the atlas to be scaled to each participant’s head size via AtlasViewer toolbox (Aasted et al., 2015). The probe was registered to the participant’s head surface and projected to the brain atlas allowing for estimation of MNI coordinate locations for each channel (i.e., represent the midpoint location of the channel or halfway between the source-detector pair). Channels were then assigned to the following regions of interest (ROIs) using their average MNI coordinate location and checked through visual inspection of their projection to the generic head atlas: superior frontal (SFG), middle frontal (MFG), inferior frontal (pars triangularis; IFGtri and pars opercularis; IFGoper), precentral (PCG), middle temporal, supramarginal (SMG), and angular gyri (AG). Average channel locations were similar across the three groups (and to that of the generic probe), and thus, the same channel assignment to ROIs was used across the participant groups. As described in greater detail in section “fNIRS Data Analysis” and consistent with methods of previous work (Li et al., 2020), data from channels that spanned the same region based on their MNI coordinate locations were averaged together to represent activation from that region or ROI (e.g., channels 1 and 2 covered left SFG and therefore, data from these channels were averaged and referred to as activation in the left SFG ROI). See Table 2 for channel assignment to ROI based on MNI coordinate location, Supplementary Table 2 for the number of participants that contributed data to each ROI, and Supplementary Table 3 for MNI coordinate locations for the midpoint of each channel, including Brodmann areas to support comparison of findings with future studies.

**TABLE 2:** Channels assigned to regions of interest.

ROI	Left hemisphere	Right hemisphere
Superior frontal gyrus (SFG)	1, 2	29, 30
Middle frontal gyrus (MFG)	4, 6, 7, 10	32, 34, 35, 38
Inferior frontal gyrus – pars triangularis (IFGtri)	9, 11, 12, 15	37, 39, 40, 43
Inferior frontal gyrus – pars opercularis (IFGoper)	13, 16	41, 44
Precentral gyrus (PCG)	18	46
Middle temporal gyrus (MTG)	19, 22, 23, 26	47, 50, 51, 54
Supramarginal gyrus (SMG)	21, 24	49, 52
Angular gyrus (AG)	25, 28	53, 56

### Management of the Lesion

Structural imaging was obtained for participants with stroke.^6^ Lesion maps were drawn manually using their structural images in MRIcron/MRIcroGL (Rorden and Brett, 2000; Fridriksson et al., 2007) and both were then normalized using SPM12 software^7^. For each participant with stroke, MNI coordinate channel locations were compared to their normalized lesion map. Any channels in areas of frank lesion were manually excluded from the analysis at the individual participant level to minimize the contribution of spurious signal from the lesion. See Supplementary Table 4 for additional detail on ROIs affected by lesion damage.

### fNIRS Data Analysis

All fNIRS data were analyzed in Homer2 (Huppert et al., 2009). The processing stream included (1) pruning channels with OD lower than 60 dB (i.e., low signal-to-noise ratio) or higher than 140 dB (i.e., saturated signal); (2) transforming raw fNIRS to optical density (OD); (3) applying an automated motion detection and correction method to the OD data (i.e., hybrid of the spline interpolation and Savitzky–Golay filtering methods; (Jahani et al., 2018); (4) low band-pass filtering the data at 0.50 Hz to remove high-frequency noise (e.g., cardiac signal) and high band-pass filtering the data at 0.01 Hz to remove low-frequency noise (e.g., drift); (5) converting changes in OD to concentration changes in HbO and HbR using the modified Beer-Lambert Law (Kocsis et al., 2006) with a differential pathlength factor of 6 (Duncan et al., 1996); and (6) estimating HRF using a general linear model (GLM) with ordinary least squares. This last step took advantage of the high temporal resolution of fNIRS and applied a sequence of consecutive Gaussian functions (i.e., standard deviations of 1 s, means separated by 1 s over the time range for one trial/block) to model the shape of the HRF as opposed to using a predetermined canonical HRF (Diamond et al., 2006; Gagnon, 2011; Jahani et al., 2017).

HRF time ranges were set in the GLM according to the task design. For the event-related semantic feature verification task, the time range was set for –2 s prior to stimulus onset to 12 s after stimulus onset (i.e., 2 s for baseline, 5 s for one trial, 7 s for return to baseline; Rosen et al., 1998). For the block design picture naming task, the time range was set for −2 s prior to stimulus onset to 20 s after stimulus onset (i.e., 2 s for baseline, 15 s for five 3-s long trials, 5 s for return to baseline). For the block-design arithmetic task, the time range was set for −2 s prior to stimulus onset and 37 s after stimulus onset (i.e., 2 s for baseline, 32 s for eight 4-s long trials, 5 s for return to baseline).^8^

Finally, global systemic physiology was regressed out from brain signal using short-separation channel regression. For every long-separation channel, the short-separation channel signal that was most highly correlated with that particular long-separation channel was used as a regressor within the GLM to remove unwanted signals detected from the scalp. This step provided a more precise estimate of the evoked HRF by reducing the influence of systemic physiology on the signal (Gagnon, 2011; Gagnon et al., 2012; Yücel et al., 2015). To further support statistical power and data quality, all participants included in the GLM had two runs of usable data; and all healthy control data had fewer than 50% of channels pruned during the first step of the processing stream detailed above. Participants in the stroke group were not excluded in this manner, given the already modest sample size.

For each ROI, mean HbO and HbR concentration change was obtained by averaging across both runs for each participant and then, across channels assigned to a particular ROI (Li et al., 2020). Paired *t*-tests were then conducted at each timepoint across the entire HRF timecourse (i.e., –2 to 12 s for semantic feature, –2 to 20 s for picture naming, –2 to 37 s for arithmetic) for each ROI to identify statistically significant differences in mean HbO and HbR concentration change between tasks conditions for each participant group as both were considered a metric of task-related neural activity. *P*-values were adjusted for multiple comparisons based on the number of ROIs (Singh and Dan, 2006; Poldrack, 2007; Poldrack and Mumford, 2009) using the Benjamini–Hochberg procedure with a FDR of 0.05 (Benjamini and Hochberg, 1995). Significant findings at the *p* < 0.05 level that did not survive FDR-adjustment were interpreted and discussed given the exploratory nature of this pilot study.

## Results

### Behavioral Data

#### Semantic Feature

**FIGURE 3:**
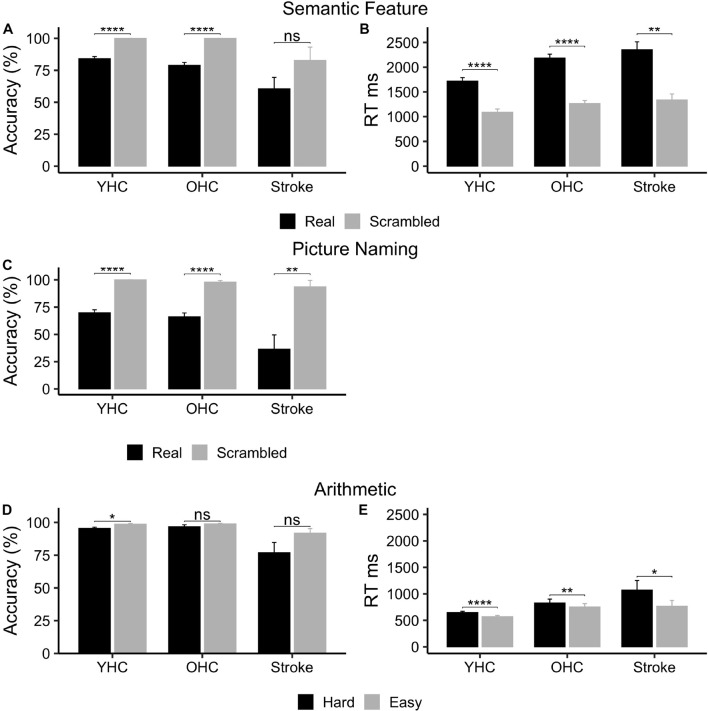
Behavioral task performance for all three participant groups. **(A)** Semantic feature – accuracy. **(B)** Semantic feature – reaction time. **(C)** Picture naming – accuracy. **(D)** Arithmetic – accuracy. **(E)** Arithmetic – reaction time. ms, milliseconds; %, percent accurate. * represents significant difference between conditions at *p* < 0.05 level, ** represents significant difference between conditions at *p* < 0.01 level, and **** represents significant difference between conditions at *p* < 0.001 level.

While all three groups demonstrated higher mean accuracy when judging features of scrambled pictures versus real pictures (Figure 3A), only the healthy control participant groups showed statistically significant differences between task conditions [Young Healthy Controls: *t*(16) = –9.33, *p* < 0.001, adj. *p* < 0.001; Older Healthy Controls: *t*(16) = –8.94, *p* < 0.001, adj. *p* < 0.001; Individuals with Stroke: *t*(5) = –1.86, *p* = 0.122, adj. *p* = 0.122]. However, statistically significant differences in performance between real pictures and scrambled pictures (Figure 3B) were found for all three groups in mean reaction time [i.e., shorter reaction times in the scrambled relative to the real picture condition; Young Healthy Controls: *t*(16) = 11.9, *p* < 0.001, adj. *p* < 0.001; Older Healthy Controls: *t*(16) = 21.8, *p* < 0.001, adj. *p* < 0.001; Individuals with Stroke: *t*(5) = 5.02, *p* = 0.004, adj. *p* = 0.004].

#### Picture Naming

As demonstrated in Figure 3C, all three participant groups were significantly more accurate when saying “skip” in response to scrambled pictures than when naming real pictures [Younger Healthy Controls: *t*(16) = –11.0, *p* < 0.001, adj. *p* < 0.001; Older Healthy Controls: *t*(14) = –8.01, *p* < 0.001, adj. *p* < 0.001; Individuals with Stroke *t*(4) = –6.17, *p* = 0.009, adj. *p* = 0.009].

#### Arithmetic

While all three participant groups were more accurate performing easy addition than hard addition problems (Figure 3D), only young healthy controls demonstrated a statistically significant difference between task conditions [Young Healthy Controls: *t*(13) = –3.18, *p* = 0.007, adj. *p* = 0.022; Older Healthy Controls: *t*(10) = –1.36, *p* = 0.204; Individuals with Stroke: *t*(3) = –2.36, *p* = 0.0998, adj. *p* = 0.150]. Yet, similar to the semantic feature verification task, statistically significant differences in performance across task conditions (Figure 3E) were found for all three groups in mean reaction time [i.e., shorter reaction times in the easy versus hard addition condition; Younger Healthy Controls: *t*(13) = 6.40, *p* < 0.001, adj. *p* < 0.001; Older Healthy Controls: *t*(10) = 3.58, *p* = 0.005; adj. *p* = 0.008; Individuals with Stroke: *t*(3) = 3.94, *p* = 0.029, adj. *p* = 0.029]. See Supplementary Table 5 for mean accuracy and reaction time by group and task.

### fNIRS Data

#### Semantic Feature

**FIGURE 4:**
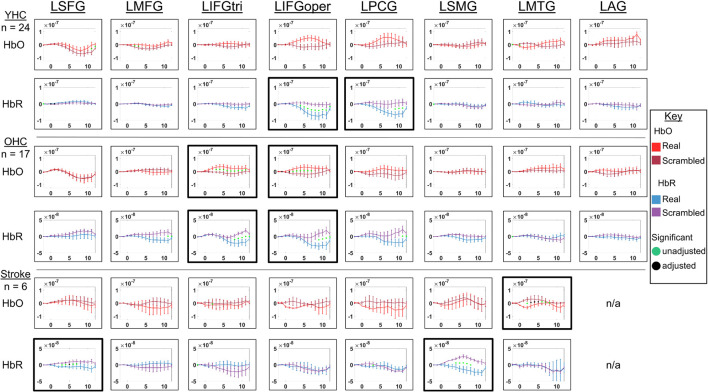
Comparison of group average HRF between task conditions for left hemisphere ROIs during semantic feature judgment. HbO and HbR changes in μM units. Significant difference between task conditions at *p* < 0.05 level are reflected by a green dot and after FDR-adjustment with alpha of 0.05 by a black dot. Black borders denote ROIs for which there was a significant difference between the task conditions for at least three consecutive seconds. When the red line is higher than the maroon line, it suggests there was greater HbO concentration change in the real than scrambled picture condition. When the blue line is lower than the purple line, it suggests there was lower HbR concentration change in the real than scrambled picture condition. Both patterns are consistent with greater neural activation (i.e., increase in oxygenated blood, decrease in deoxygenated blood) in the real than scrambled picture condition and vice versa. LSFG, left superior frontal gyrus; LMFG, left middle frontal gyrus; LIFGtri, left inferior frontal gyrus pars triangularis; LIFGoper, left inferior frontal gyrus pars opercularis; LPCG, left precentral gyrus; LSMG, left supramarginal gyrus; LAG, left angular gyrus; YHC, younger healthy controls; OHC, older healthy controls; HbO, oxygenated hemoglobin concentration change; HbR, deoxygenated hemoglobin concentration change, N/A, not available due to combination of pruned channels due to poor SNR and lesion.

**FIGURE 5:**
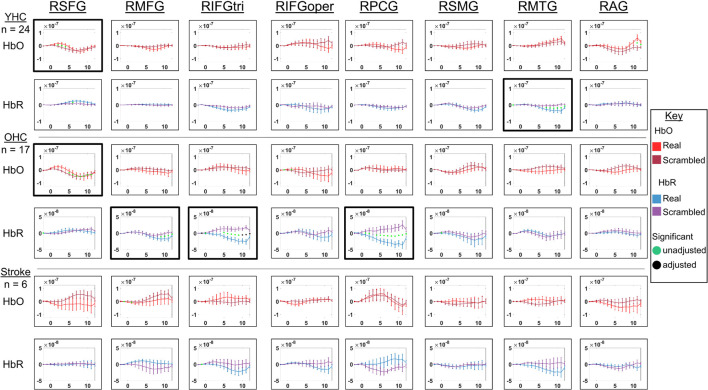
Comparison of group average HRF between task conditions for right hemisphere ROIs during semantic feature judgment. HbO and HbR changes in μM units. Significant difference between task conditions at *p* < 0.05 level are reflected by a green dot and after FDR-adjustment with alpha of 0.05 by a black dot. Black borders denote ROIs for which there was a significant difference between the task conditions for at least three consecutive seconds. When the red line is higher than the maroon line, it suggests there was greater HbO concentration change in the real than scrambled picture condition. When the blue line is lower than the purple line, it suggests there was lower HbR concentration change in the real than scrambled picture condition. Both patterns are consistent with greater neural activation (i.e., increase in oxygenated blood, decrease in deoxygenated blood) in the real than scrambled picture condition and vice versa. RSFG, right superior frontal gyrus; RMFG, right middle frontal gyrus; RIFGtri, right inferior frontal gyrus pars triangularis; RIFGoper, right inferior frontal gyrus pars opercularis; RPCG, right precentral gyrus; RSMG, right supramarginal gyrus; RAG, right angular gyrus; YHC, younger healthy controls; OHC, older healthy controls; HbO, oxygenated hemoglobin concentration change; HbR, deoxygenated hemoglobin concentration change.

Full results of significance tests are available in Figures 4, 5.

##### Left hemisphere

*HbO* As shown in Figure 4, young healthy controls demonstrated no significant differences in HbO concentration change when judging features of real versus scrambled pictures; older healthy controls showed significantly higher HbO concentration change when judging features of real versus scrambled pictures in LIFGtri and LIFGoper; and individuals with post-stroke aphasia showed significantly higher HbO concentration change when judging features of scrambled versus real pictures in LMTG.

*HbR* As demonstrated in Figure 4, young healthy controls exhibited significantly greater HbR concentration change when judging features of real versus scrambled pictures in LIFGoper and LPCG; older healthy controls showed significantly higher HbR concentration change when judging features of real versus scrambled pictures in LIFGtri and LIFGoper; and individuals with aphasia showed significantly higher HbR concentration change when judging features of real versus scrambled pictures in LSFG and LSMG.

##### Right hemisphere

*HbO* As shown in Figure 5, both younger and older healthy controls demonstrated significantly higher HbO concentration change when judging features of real versus scrambled pictures in RSFG; and individuals with stroke showed no significant differences in HbO concentration change between the real and scrambled picture conditions.

*HbR* As demonstrated in Figure 5, younger healthy controls demonstrated significantly lower HbR concentration change when judging features of real versus scrambled pictures in RMTG; older healthy controls showed significantly lower HbR concentration change when judging features of real versus scrambled pictures in RMFG, RIFGtri, and RPCG; and individuals with aphasia showed no significant HbR concentration changes between the real and scrambled picture condition, consistent with their pattern in HbO in the right hemisphere.

**FIGURE 6:**
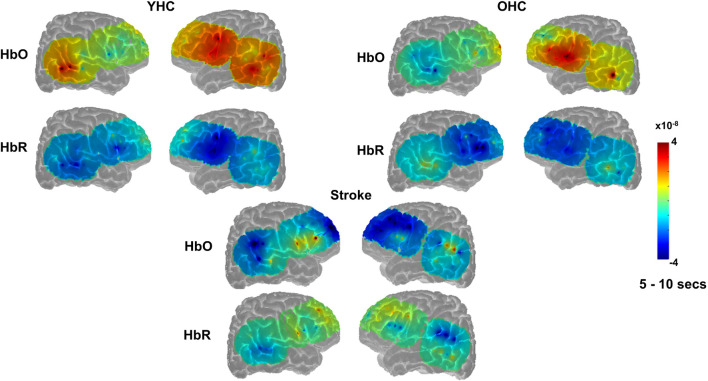
Group mean HRF concentration overlays displaying the real minus scrambled picture contrast during semantic feature judgment. The color bar reflects the scale of the concentration change in μM units. HbO, oxygenated hemoglobin concentration change; HbR, deoxygenated hemoglobin concentration change; YHC, younger healthy controls; OHC, older healthy controls.

Overall, Figures 4, 5 demonstrate that activation differences between the real and scrambled semantic feature judgment conditions across groups generally emerged between 5 and 10 s and predominantly in HbR. Figure 6 provides the group mean HbO and HbR concentration overlays (i.e., interpolation of the results across the brain surface, which differs from a statistical T-map) for the real minus scrambled picture condition during the 5–10 s time range. At a high-level, Figure 6 reveals that younger healthy controls engaged the left-lateralized language network with some activation in right middle temporal gyrus; older healthy controls showed similar activation patterns to the younger group with the exception of the right temporal activation, and individuals with stroke exhibited lower overall signal amplitude than the other two groups with some engagement of right middle/inferior frontal regions during real versus scrambled picture judgment.

#### Picture Naming

**FIGURE 7:**
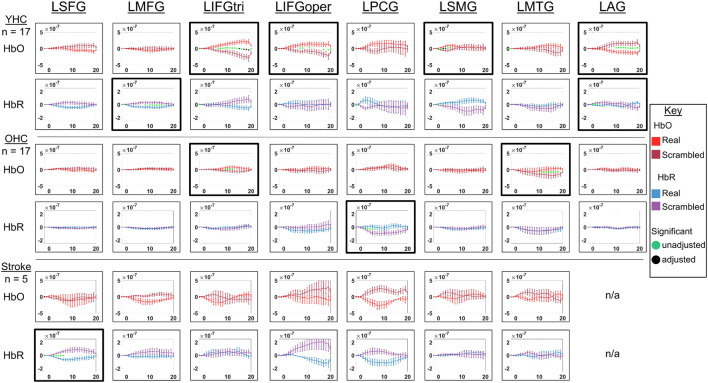
Comparison of group average HRF between task conditions for left hemisphere ROIs during picture naming. HbO and HbR changes in μM units. Significant difference between task conditions at *p* < 0.05 level are reflected by a green dot and after FDR-adjustment with alpha of 0.05 by a black dot. Black borders denote ROIs for which there was a significant difference between the task conditions for at least three consecutive seconds. When the red line is higher than the maroon line, it suggests there was greater HbO concentration change in the real than scrambled picture condition. When the blue line is lower than the purple line, it suggests there was lower HbR concentration change in the real than scrambled picture condition. Both patterns are consistent with greater neural activation (i.e., increase in oxygenated blood, decrease in deoxygenated blood) in the real than scrambled picture condition and vice versa. LSFG, left superior frontal gyrus; LMFG, left middle frontal gyrus; LIFGtri, left inferior frontal gyrus pars triangularis; LIFGoper, left inferior frontal gyrus pars opercularis; LPCG, left precentral gyrus; LSMG, left supramarginal gyrus; LAG, left angular gyrus; YHC, younger healthy controls; OHC, older healthy controls; HbO, oxygenated hemoglobin concentration change; HbR, deoxygenated hemoglobin concentration change; N/A, not available due to combination of pruned channels due to poor SNR and lesion.

**FIGURE 8:**
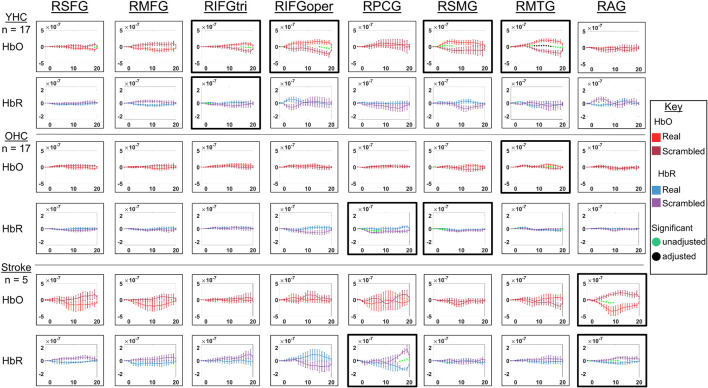
Comparison of group average HRF between task conditions for right hemisphere ROIs during picture naming. HbO and HbR changes in μM units. Significant difference between task conditions at *p* < 0.05 level are reflected by a green dot and after FDR-adjustment with alpha of 0.05 by a black dot. Black borders denote ROIs for which there was a significant difference between the task conditions for at least three consecutive seconds. When the red line is higher than the maroon line, it suggests there was greater HbO concentration change in the real than scrambled picture condition. When the blue line is lower than the purple line, it suggests there was lower HbR concentration change in the real than scrambled picture condition. Both patterns are consistent with greater neural activation (i.e., increase in oxygenated blood, decrease in deoxygenated blood) in the real than scrambled picture condition and vice versa. RSFG, right superior frontal gyrus; RMFG, right middle frontal gyrus; RIFGtri, right inferior frontal gyrus pars triangularis; RIFGoper, right inferior frontal gyrus pars opercularis; RPCG, right precentral gyrus; RSMG, right supramarginal gyrus; RAG, right angular gyrus; YHC, younger healthy controls; OHC, older healthy controls; HbO, oxygenated hemoglobin concentration change; HbR, deoxygenated hemoglobin concentration change.

Full results of significance tests are available in Figures 7, 8.

##### Left hemisphere

*HbO* As depicted in Figure 7, younger healthy controls showed significantly higher HbO concentration change in LIFGtri, LIFGoper, and LSMG when naming real pictures versus producing “skip” in response to scrambled pictures. This group also demonstrated significantly higher HbO concentration change in the scrambled than real picture condition in LAG, consistent with previous neuroimaging work suggesting this region may serve automatic processing functions (e.g., Humphreys and Lambon Ralph, 2015), a point that will be returned to in the discussion. Older healthy controls demonstrated significantly higher HbO concentration change in the real than scrambled picture condition in LIFGtri and LMTG. Finally, there were no significant differences in HbO concentration change between the real and scrambled picture conditions in any of the left hemisphere ROIs for the stroke group.

*HbR* As represented in Figure 7, young healthy controls demonstrated significantly lower HbR concentration change when naming real pictures versus producing “skip” in response to scrambled pictures in LMFG and LAG. Older healthy controls showed significantly lower HbR concentration change in the scrambled than real picture condition in LPCG. Individuals with stroke exhibited significantly lower HbR concentration change in the real versus scrambled picture condition in LSFG.

##### Right hemisphere

*HbO* As depicted in Figure 8, younger healthy controls showed significantly higher HbO concentration when naming real pictures versus producing “skip” in response to scrambled pictures change in RIFGtri, RIFGoper, RSMG, and RMTG. Older healthy controls showed significantly higher HbO concentration change in RMTG in the real than scrambled picture condition, similar to young healthy controls. Finally, individuals with stroke showed significantly higher HbO concentration change in the scrambled than real condition in RAG. This pattern is similar to what young healthy controls demonstrated in the left hemisphere homologue of this region (i.e., LAG, see Figure 8) in the scrambled versus real condition for this task—an area that was damaged in the stroke group. Although it must be interpreted with caution given the sample size, this finding is consistent with expectations for post-stroke brain reorganization (e.g., functions previously performed by damaged left hemisphere may be performed by right hemisphere homologue) and will be returned to in the discussion.

*HbR* As represented in Figure 8, young healthy controls did not show significantly lower HbR concentration change in the real versus scrambled condition for any of the right hemisphere ROIs. However, they did exhibit significantly lower HbR concentration change when producing “skip” in response to scrambled pictures compared to when naming real pictures in RIFGtri. Older healthy controls showed significantly lower HbR concentration change in the scrambled than real pictures condition in RPCG and RSMG. Individuals with stroke exhibited significantly lower HbR concentration change in the real than scrambled condition in RPCG and RAG.

**FIGURE 9:**
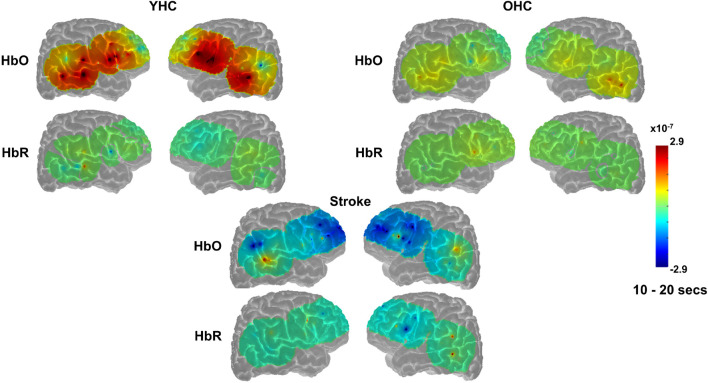
Group mean HRF concentration overlays displaying the real minus scrambled picture contrast during picture naming. The color bar reflects the scale of the concentration change in μM units. HbO, oxygenated hemoglobin concentration change; HbR, deoxygenated hemoglobin concentration change; YHC, younger healthy controls; OHC, older healthy controls.

Overall, Figures 7, 8 reveal that activation differences between the real and scrambled picture naming conditions across groups tended to occur between 10 and 20 s and to a greater extent in HbO. Figure 9 provides the group mean HbO and HbR concentration overlays for the real minus scrambled picture condition during the 10–20 s time range. At a summary level, Figure 9 reflects that younger healthy controls engaged bilateral perisylvian and middle temporal regions when naming real pictures versus producing “skip” in response to scrambled pictures; older healthy controls showed similar activation patterns to the younger group but at a lower signal amplitude, and individuals with stroke recruited right middle temporal gyrus, which is reasonable in the context of left hemisphere damage in that group.

#### Arithmetic

**FIGURE 10:**
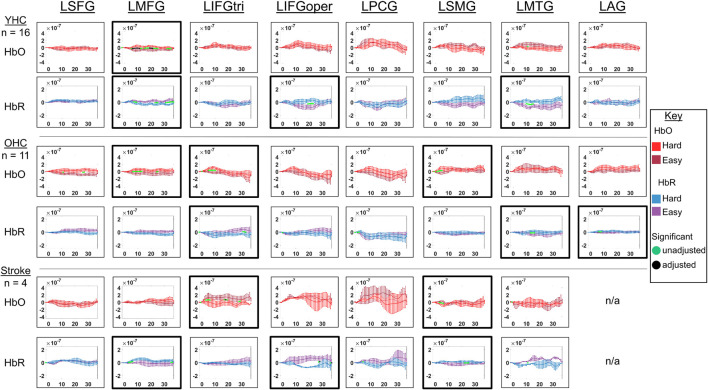
Comparison of group average HRF between task conditions for left hemisphere ROIs during hard vs. easy addition. HbO and HbR changes in μM units. Significant difference between task conditions at *p* < 0.05 level are reflected by a green dot and after FDR-adjustment with alpha of 0.05 by a black dot. Black borders denote ROIs for which there was a significant difference between the task conditions for at least three consecutive seconds. When the red line is higher than the maroon line, it suggests there was greater HbO concentration change in the hard than easy addition condition. When the blue line is lower than the purple line, it suggests there was lower HbR concentration change in the hard than easy addition condition. Both patterns are consistent with greater neural activation (i.e., increase in oxygenated blood, decrease in deoxygenated blood) in the hard than easy addition and vice versa. LSFG, left superior frontal gyrus; LMFG, left middle frontal gyrus; LIFGtri, left inferior frontal gyrus pars triangularis; LIFGoper, left inferior frontal gyrus pars opercularis; LPCG, left precentral gyrus; LSMG, left supramarginal gyrus; LAG, left angular gyrus; YHC, younger healthy controls; OHC, older healthy controls; HbO, oxygenated hemoglobin concentration change; HbR, deoxygenated hemoglobin concentration change; N/A, not available due to combination of pruned channels due to poor SNR and lesion.

**FIGURE 11:**
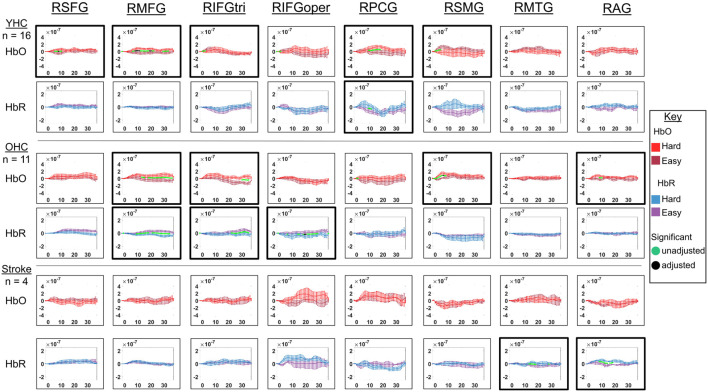
Comparison of group average HRF between task conditions for right hemisphere ROIs during hard vs. easy addition. HbO and HbR changes in μM units. Significant difference between task conditions at *p* < 0.05 level are reflected by a green dot and after FDR-adjustment with alpha of 0.05 by a black dot. Black borders denote ROIs for which there was a significant difference between the task conditions for at least three consecutive seconds. When the red line is higher than the maroon line, it suggests there was greater HbO concentration change in the hard than easy addition condition. When the blue line is lower than the purple line, it suggests there was lower HbR concentration change in the hard than easy addition condition. Both patterns are consistent with greater neural activation (i.e., increase in oxygenated blood, decrease in deoxygenated blood) in in the hard than easy addition condition and vice versa. RSFG, right superior frontal gyrus; RMFG, right middle frontal gyrus; RIFGtri, right inferior frontal gyrus pars triangularis; RIFGoper, right inferior frontal gyrus pars opercularis; RPCG, right precentral gyrus; RSMG, right supramarginal gyrus; RAG, right angular gyrus; YHC, younger healthy controls; OHC, older healthy controls; HbO, oxygenated hemoglobin concentration change; HbR, deoxygenated hemoglobin concentration change.

Full results of significance tests are available in Figures 10, 11.

##### Left hemisphere

*HbO* As shown in Figure 10, young healthy controls showed higher HbO concentration change when solving hard versus easy addition problems in LMFG. Older healthy controls demonstrated higher HbO concentration change in the hard than easy addition condition in LMFG, LIFGtri, and LSMG. Individuals with stroke showed significantly higher HbO concentration change in the easy than hard addition condition in LIFGtri and LSMG.

*HbR* As revealed in Figure 10, young healthy controls showed significantly lower HbR concentration change in the hard than easy addition condition in LMFG and in the easy than hard addition condition in LIFGoper and LMTG. Older healthy controls showed significantly lower HbR concentration change during the hard than easy addition condition in LIFGtri. Similar to young healthy controls, older controls also showed significantly lower HbR concentration change during the easy than hard addition condition in LMTG in addition to LAG. Individuals with stroke demonstrated significantly lower HbR concentration change in the hard than easy addition condition in LIFGoper and in the easy than hard addition condition in LMFG and LSMG.

##### Right hemisphere

*HbO* As depicted in Figure 11, younger healthy controls showed significantly higher HbO concentration change in the hard than easy addition condition in RSFG, RMFG, and RPCG and in the easy than hard addition condition in RIFGtri and RSMG. Older healthy controls exhibited higher HbO concentration change during the hard versus easy addition condition in RMFG, RIFGtri, RSMG, and RAG. Individuals with stroke demonstrated no significant differences in HbO concentration change between arithmetic task conditions in any of the right hemisphere ROIs.

*HbR* As demonstrated in Figure 11, younger healthy controls showed significantly higher HbR concentration change in the easy than hard addition condition in RPCG. Older healthy controls showed significantly lower HbR concentration change in the hard versus easy addition condition in RMFG, RIFGtri, and RIFGoper. Individuals with stroke showed significantly lower HbR concentration change in the easy than hard addition condition in RMTG and RAG.

**FIGURE 12:**
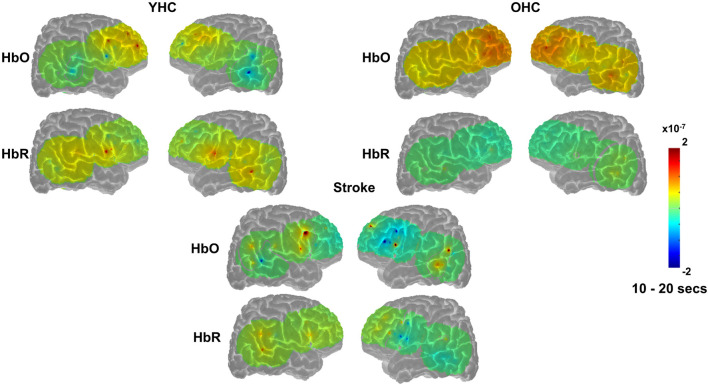
Group mean HRF concentration overlays displaying the hard minus easy addition contrast during the arithmetic task. The color bar reflects the scale of the concentration change in μM units. HbO, oxygenated hemoglobin concentration change; HbR, deoxygenated hemoglobin concentration change; YHC, younger healthy controls; OHC, older healthy controls.

Overall, Figures 10, 11 show that activation differences between the hard and easy addition conditions across groups tended to occur between 10 and 20 s with some exceptions (e.g., RSMG in young healthy, RIFGtri in older healthy, LSMG in individuals with aphasia) and differences were evident in both HbO and HbR. Figure 12 provides the group mean HbO and HbR concentration overlays for the hard minus easy addition condition during the 10–20 s time range. Broadly, Figure 12 shows that younger healthy controls engaged bilateral middle frontal gyri when solving hard addition problems compared to easy addition problems; older healthy also recruited bilateral middle frontal gyri in addition to demonstrating higher signal amplitude than both groups across the brain (i.e., warm colors in HbO), and individuals with stroke showed some engagement of the domain-general network (i.e., bilateral IFGoper).

## Discussion

This study assessed activation patterns in neurotypicals and post-stroke individuals during language and domain-general cognitive task processing using fNIRS and provided several important findings. First, in line with the hypotheses, healthy controls demonstrated activation in core areas of the left-lateralized language network (Wilson and Schneck, 2020) when judging semantic features (i.e., younger healthy controls: LIFGoper; older healthy controls: LIFGtri, LIFGoper) or naming pictures (i.e., younger healthy controls: LIFGtri, LIFGoper; older healthy controls LIFGtri, LMTG) and in the domain-general network when solving hard relative to easy addition problems in both younger and older healthy controls (i.e., bilateral MFG). Second, when activation patterns were qualitatively compared between the healthy control groups, older healthy controls demonstrated more extensive right hemisphere engagement during the semantic feature task (i.e., RSFG, RMFG, RIFGtri, RPCG) than younger healthy controls (i.e., RSFG, RMTG) as hypothesized based on previous neuroimaging studies investigating age-related changes in language function. However, younger healthy controls demonstrated more extensive right hemisphere engagement during the picture naming task (i.e., RIFGtri, RIFGoper, RSMG, RMTG) than older healthy controls (i.e., RMTG), which was not anticipated. Third, some ROIs (e.g., LAG in young healthy controls and RAG in individuals with aphasia during picture naming) showed greater engagement during the control than the experimental condition, highlighting the complexity of brain-behavior relationships and nuanced roles of some brain regions. Finally, in regards to findings in the post-stroke aphasia group, fewer ROIs showed significant differences in HRF between the conditions, which may have been a factor of the sample size in addition to the increased variability (i.e., low signal-to-noise ratio) and/or other alterations in the hemodynamic response in this population (Bonakdarpour et al., 2007). During the language tasks, individuals with post-stroke aphasia also demonstrated significant activation in areas outside the traditional language network and different regions than those engaged by healthy controls (i.e., semantic: LSFG, LSMG; PN: LSFG, RPCG)— a reasonable finding in the context of left perisylvian damage (Kiran et al., 2019).

This study uniquely contributed to the field of fNIRS and stroke-induced aphasia in several ways: (1) implementation of a novel method to manage lesioned brain areas when analyzing fNIRS data collected from individuals with stroke-induced aphasia; (2) acquisition of fNIRS data via a multi-channel probe that spanned widespread aspects of the language network (i.e., bilateral frontal, temporal, parietal regions) in individuals with aphasia; and (3) application of short-separation channel regression in the GLM to minimize the influence of global systemic physiology on the evoked brain signal collected from individuals with aphasia. This comprehensive and methodologically rigorous approach has not been taken in previous studies using fNIRS to investigate language processing in individuals with stroke-induced aphasia. Sakatani et al. (1998) acquired data from intact left prefrontal cortex only. Similarly, Hara et al. (2017) captured signal from a narrow aspect of the language network (i.e., bilateral STG an IFG), which was spared in all participants. Neither of those studies reported to have employed strategies to reduce the influence of global systemic physiology on the evoked brain signal as in the present work. Thus, this pilot study will serve as a crucial stepping stone for future fNIRS studies in aphasia, including investigating neural mechanisms of successful language performance in individuals with aphasia during ecologically valid tasks (e.g., retelling a recent experience to a conversation partner seated next to them) and conducting routine monitoring of neural mechanisms supporting treatment-related language recovery (e.g., repeated fNIRS measurements conducted in the clinician’s office over the course of therapy—a safe and affordable option, not feasible with fMRI). These points in addition to key results will be discussed in greater detail in the paragraphs that follow.

### Activation Patterns During Semantic Processing and Comparison to Previous Work

Young healthy controls activated areas of the language network in the left (i.e., LIFGoper, LPCG) and right hemisphere (i.e., RMTG) in addition to anterior, superior frontal cortex (i.e., RSFG) when judging semantic features of real pictures. In older healthy controls, activation was more widespread, showing recruitment of left IFG pars triangularis and opercularis and right SFG, MFG, IFG pars triangularis, and PCG. This finding appears more bilateral and less left-lateralized than previous fMRI studies using this imaging task (i.e., LSFG, LMFG, LIFGtri, BLoper, LPCG; Kiran et al., 2015; Meier et al., 2018, 2019). Additionally, older healthy controls in previous studies activated bilateral MTG and AG when judging semantic features of real pictures—key areas for semantic processing (Binder et al., 2009) that were not significantly engaged by older healthy controls in the present study. This difference does not appear to be due to the posterior location of these ROIs that were more susceptible to pruning due to poor signal-to-noise ratio associated with increased density of hair, as most participants contributed data to these ROIs (see Supplementary Table 2). Rather, it is more plausible that the activation for some regions in previous studies was located in aspects of those regions not captured by this study’s probe (e.g., anterior or middle aspects of MTG; anterior/superior aspects of AG).

Only a few studies have specifically examined semantic processing in healthy individuals using fNIRS (Kennan et al., 2002; Noguchi et al., 2002; Amiri et al., 2014). Thus, the present study’s findings provide unique insights into the utility of fNIRS to answer questions about the neural bases of this linguistic process. For example, Kennan et al. (2002) demonstrated that young healthy controls more heavily relied on the left (than the right) inferior frontal cortex to perform semantic processing. At a high-level, group mean HbO concentration change overlay figures showing the real minus scrambled picture contrast for the young healthy control group in this study agree with the findings from Kennan et al. (2002). However, upon closer inspection of the ROI activation results, there is right hemisphere involvement during this task in the young healthy controls (i.e., RSFG, RMTG) not seen in the Kennan study. Kennan et al. (2002) measured bilateral inferior frontal gyri in young healthy controls whereas the current study assessed bilateral frontal, temporal, and parietal regions. In the present study, young healthy controls showed anterior (i.e., RSFG) and posterior (i.e., RMTG) right hemisphere activation, which may explain the reduced extent of findings in the right hemisphere reported in the Kennan study and emphasizes a unique contribution of the present work. Importantly, the findings of the present study aligned well with Amiri et al. (2014) in that older individuals in that study, which applied both continuous-wave and time-domain fNIRS, also showed activation in bilateral dorsolateral prefrontal, frontotemporal, and inferior parietal areas. This convergence is encouraging in the face of concern that the present study’s findings in the older healthy control group may have been limited by the use of continuous-wave fNIRS (i.e., relative assessments of HRF with continuous-wave fNIRS could be influenced by increases in scalp-to-cortex distance with age). Overall, these findings build on prior work and emphasize the importance of using a whole-brain approach to study complex linguistic processes, like semantics.

### Activation Patterns During Picture Naming and Comparison to Previous Work

Young healthy controls demonstrated significant activation in left hemisphere language areas (i.e., IFGtri, IFGoper, SMG), right hemisphere language area homologues (i.e., IFGtri, IFGoper, SMG, MTG) and aspects of the domain-general network (i.e., LMFG) during lexical retrieval. As discussed previously, a number of studies have also investigated lexical retrieval in young healthy individuals using fNIRS (e.g., Cannestra et al., 2003; Zhang et al., 2017). In agreement with prior fNIRS studies (Cannestra et al., 2003; Zhang et al., 2017), the young healthy control group in the present study recruited LIFGtri during picture naming. However, Cannestra et al. (2003) also found deactivation in LPCG, which was not demonstrated in the current study. Given the engagement of LPCG for articulation, this difference in findings may have been related to Cannestra et al.’s (2003) use of covert naming. Finally, as will be returned to later in the discussion, some groups have emphasized the utility of using HbR concentration change as a proxy for activation as opposed to HbO, and in particular when fNIRS tasks involve overt speaking (Zhang et al., 2017; Hirsch et al., 2018; Descorbeth et al., 2020). However, activation results in the present study based on HbR concentration change did not appear to align more closely with prior fMRI work conducted with this overt picture naming task than HbO concentration change. Finally, the older healthy control group in the present study relied on left IFGtri, left MTG, and right MTG during picture naming; however, in prior work by Kiran and colleagues this age group recruited a wider swath of regions, including SFG, IFGtri, and PCG in the left hemisphere and MFG, IFGoper, MTG and SMG bilaterally (Sebastian and Kiran, 2011; Kiran et al., 2015). Future work using time-domain fNIRS and/or high-density whole-brain arrays with a larger sample of older healthy controls will be necessary to determine if this divergence in findings is replicable and if so, to identify the underlying mechanism.

### Activation Patterns During Domain-General Processing and Comparison to Previous Work

During the hard versus easy addition contrast of the arithmetic task, both control groups and individuals with aphasia activated aspects of the domain-general network (i.e., bilateral MFG, RPCG in young healthy; bilateral MFG, LSMG, RIFGoper, RSMG, RAG in older healthy; LIFGoper in post-stroke aphasia) as hypothesized based on previous findings in fMRI (Fedorenko et al., 2013; Blank et al., 2015). Some areas were inconsistent though (e.g., LMTG activation in young healthy controls; bilateral IFGtri in older healthy controls) and warrant further discussion. The young healthy control group findings (i.e., increased LMFG activation in the hard vs. easy addition contrast accompanied by increased LMTG activation in the easy vs. hard addition) were overall consistent with findings from previous fNIRS studies examining neural correlates of arithmetic difficulty in healthy young individuals (i.e., increased left inferior frontal activation and decreased inferior parietal lobule and RMTG activation in hard versus easy arithmetic contrast; Artemenko et al., 2018, 2019). The bilateral IFGtri engagement seen in older healthy controls is in agreement with work suggesting that this age group may recruit bilateral cortex to compensate for age-related neural decline and maintain task accuracy. It is also possible that the bilateral activation in LIFGtri reflects domain-general processing, given research showing that adjacent aspects of Broca’s area can serve different functions (i.e., language and domain-general cognitive control; Fedorenko et al., 2012).

### Activation Patterns in Individuals With Post-stroke Aphasia

Individuals with post-stroke aphasia demonstrated activation in fewer ROIs than the younger and older healthy control groups across tasks. As mentioned previously, this finding may have been due to low power and/or increased variability in the hemodynamic response in this group (Bonakdarpour et al., 2007). Significantly higher HbO concentration change was observed in the real than scrambled picture condition in left SFG in both semantic feature judgment and picture naming. Dorsolateral aspects of SFG have been implicated in attention and working memory (Li et al., 2013), suggesting that individuals with aphasia may have recruited left SFG during language processing to maintain information in short-term memory (e.g., retrieving the name of the picture while reading the written phrase during semantic feature judgment). This finding is in line with work in the field of aphasia emphasizing the role of non-linguistic cognition in language functions (Wright and Shisler, 2005; Villard and Kiran, 2017; Gilmore et al., 2019). Individuals with aphasia also demonstrated engagement of LSMG during semantic feature and RPCG during picture naming, which were not significantly engaged by the healthy groups for the real picture condition and may represent recruitment of domain-general cognitive regions when processing language in the context of aphasia (i.e., engaged when task complexity increases, irrespective of modality; Fedorenko et al., 2013). This interpretation is reasonable as language processing was indeed challenging for individuals with aphasia in this study as evidenced by real picture condition accuracy in the semantic feature and picture naming tasks of 60.53 and 36.43%, respectively. In terms of the arithmetic task, this group showed significantly higher HbO concentration change LIFGoper in the hard addition condition. This region is considered to be part of the domain-general network and was expected to be engaged during hard versus easy task processing. Nevertheless, this finding should be taken with caution as only three participants contributed data to this ROI. In sum, while the language task results were diminished relative to previous fMRI studies using these tasks with individuals with post-stroke aphasia, the findings detailed in this paragraph provide early support for the application of fNIRS to study language and domain-general cognitive in this population.

### Findings From the Control Versus Experimental Condition Contrast

Some interesting findings were revealed for the control versus experimental contrasts. In the semantic feature task, individuals with aphasia showed greater activation in LMTG when judging features of scrambled versus real pictures. This finding may be explained by difficulty completing the scrambled condition (mean accuracy: 82.72%) and thus more neural resources were needed for even the simpler aspect of the task – a pattern not seen in the healthy control groups (mean accuracy: 100%). Or, it may represent this region’s involvement in general semantic processing; Binder et al., 2009). Finally, it could be a reflection of the proximity of channels in the LMTG ROI to the inferior parietal ROIs (i.e., SMG, AG) and thereby, demonstrate activation associated with automatic processing as described in more detail in the following paragraph.

In the picture naming task, AG was recruited to a greater extent when saying “skip” in response to scrambled pictures than when naming real pictures. Young healthy controls showed significant engagement of LAG and individuals with stroke showed the same pattern in RAG. This right hemisphere activation in individuals with aphasia may reflect compensatory brain reorganization in that three of the individuals with aphasia had damage in all of the channels contributing to the LAG ROI rendering that region unavailable to serve this function. It is well known that AG is a multi-dimensional region in the brain serving a range of roles, including semantic processing, attention, reading, and calculation to name a few (Seghier, 2013). The activation in LAG demonstrated by young healthy controls and RAG in individuals with aphasia is reasonable in the context of studies suggesting its function as a temporary buffer for information requiring integration (Humphreys and Lambon Ralph, 2015; Branzi et al., 2019; Humphreys et al., 2019) and role in automatic processing (Davey et al., 2015). Further, studies have shown a task difficulty effect in AG similar to the one in the present study (i.e., activation during easy versus hard contrast when performing both less complex semantic and not semantically based decisions; see Humphreys et al., 2021 for review).

### Behavioral Task Performance

First and foremost, findings from the behavioral task performance analysis revealed that participants were indeed performing the tasks during the fNIRS measurements and largely support the interpretation of the activation results as reflecting neural engagement for the processes of interest (i.e., semantic processing, lexical retrieval, domain-general cognitive control). Participants across all three groups were more accurate and faster to respond in the less complex control condition (i.e., scrambled pictures in the language tasks, easy addition in the arithmetic task) than the more complex experimental condition (i.e., real pictures in the language tasks, hard addition in the arithmetic task), as expected, with individuals with stroke consistently demonstrating the lowest accuracy and longest reaction times across the three tasks, irrespective of condition. Although participants with aphasia did not show a statistically significant difference in accuracy between the real and scrambled conditions of the semantic feature task, they were performing above chance accuracy in both conditions, supporting that they were engaged in the task (Supplementary Table 5). However, these differences in task performance across conditions indicate that the tasks were not well-matched for cognitive complexity (Wilson and Schneck, 2020). Thus, engagement of a domain-general region (e.g., bilateral MFG in older healthy controls during the semantic task) during the language tasks may have been due to the increased complexity of performing the experimental (i.e., yes/no semantic feature judgment of a pictured object or naming a pictured object) versus the control condition (i.e., yes/no color judgment of a scrambled picture or producing “skip” in response to a scrambled picture) as opposed to pure recruitment of that system for language processing. Future work will consider the use of adaptive neuroimaging tasks (i.e., item difficulty can be varied based on participant performance; see Wilson et al., 2018 for an example) to circumvent this challenge. Matching for cognitive complexity will be especially important for studying domain-general cognitive recruitment in people with aphasia during language tasks (i.e., they may recruit these regions when language is “hard” post-stroke) and more sophisticated neuroimaging experimental designs than those employed in the current study are required to tackle the important question regarding the role of the domain-general network in language processing in people with aphasia in the future.

### Methodological Advances

This study expanded on previous fNIRS work investigating language in healthy controls and individuals with stroke in several methodological ways. First, similar to recently published work by Li et al. (2020), channels were averaged across a particular region to create an ROI, thereby increasing statistical power to detect activation in that area. Second, this study took advantage of the benefits of using a flexible GLM to model HRF (i.e., does not restrict the shape of HRF) and conducted statistical analysis across the entire timecourse as opposed to the more traditional average across a particular time range. HRF timing is not expected to be constant across ROIs. Depending on their role, some regions may engage in the task early on, while others may be recruited later in the time course. Thus, using the same time range for statistics for all ROIs can be problematic. Additionally, this decision was especially important in the current study given the need to assess HRF across three different participant groups with potentially different HRF due to variations in scalp-to-cortex distance and cerebrovascular function as a function of age and/or brain damage. Nevertheless, the superior temporal resolution of fNIRS allowed for the preliminary observation that the timing of the hemodynamic response was relatively similar across the groups in this study, although this topic deserves further exploration in a larger sample of individuals with stroke-induced aphasia. Third, as discussed throughout the manuscript, only two published studies have used fNIRS to assess language function in individuals with aphasia (Sakatani et al., 1998; Hara et al., 2017)—both of which suffered from some methodological limitations (e.g., no short-channel regression, comparison to rest versus active control condition, incomplete documentation regarding lesion management). The present study overcame these challenges by (1) including short-channel regression to remove scalp signal and increase precision of HRF estimation, (2) statistically comparing HRF in the experimental condition (e.g., hard addition) to an active control condition (e.g., easy addition), and (3) carefully managing areas of lesion in the fNIRS data analysis by comparing MNI coordinate estimates for source-detector pairs to MNI coordinates of the lesion in the post-stroke group and then excluding those in an area of frank lesion from analysis.

It is also worth highlighting that both HbO and HbR concentration changes were monitored for activation in this study, providing a thorough assessment of HRF in bilateral ROIs spanning the frontal, temporal and parietal lobe. While both an increase in HbO concentration change and a decline in HbR concentration change are expected during neural activity, there is inconsistency within the fNIRS community regarding which of these signals should be used to interpret brain activation (Pinti et al., 2019). Some experts in the field suggest reporting concentration changes in both chromophores (Pinti et al., 2019). Other researchers propose using HbO given its higher signal-to-noise ratio relative to HbR (Hoshi et al., 2001; Strangman et al., 2002; Pan et al., 2019). Finally, other experts suggest using HbR as it is less susceptible to system-level changes in physiology (e.g., blood pressure, respiration, blood flow; Wobst et al., 2001; Obrig and Villringer, 2003; Kirilina et al., 2012; Tachtsidis and Scholkmann, 2016; Zhang et al., 2017; Descorbeth et al., 2020; Dravida et al., 2020) than HbO. Further, some studies investigating overt speech production (i.e., using picture naming as in the present study) have found that HbR better distinguished between language versus motor-related activation in healthy individuals (Cannestra et al., 2003) and between groups of healthy individuals and individuals with post-stroke aphasia (Sakatani et al., 1998). As the influence of global systemic physiology on HRF (greatest concern for HbO) can be accounted for using short-channel regression (Gagnon et al., 2012; Yücel et al., 2015; Wyser et al., 2020) and both HbO and HbR have been strongly correlated with the BOLD response in fMRI (Strangman et al., 2002; Huppert et al., 2006), the present study used both HbO and HbR concentration change as metrics of neural activity. Notably, in this study, HbR appeared more sensitive to task engagement during the semantic feature task across the participant groups; HbO appeared more sensitive to task engagement during the picture naming task, and both HbO and HbR demonstrated differences between the conditions in the arithmetic task.

### Limitations and Future Directions

Nevertheless, this study was not without limitations. Some of the activation in previous fMRI studies using these tasks was captured in deeper brain structures that could not be measured without a high-density probe (e.g., activation in anterior cingulate cortex, a key region in the domain-general network could not be assessed) or aspects of superficial cortex that were not well-covered by this study’s probe (e.g., superior temporal gyrus). In a similar vein, as mentioned in the introduction, fNIRS has lower spatial resolution than fMRI (i.e., ∼10 mm vs. 1–10 mm; Quaresima and Ferrari, 2019) and thus, the interpretation of findings at the precise level of the gyri should be considered with this limitation in mind. Further, the stroke group’s sample size was relativity modest, which limited statistical power and strong interpretation of the findings in that group. Finally, as task runs were presented in consecutive order, it is possible that neural findings overall may have been influenced by an order effect. Future work replicating these findings should include counterbalanced run and block order with random presentation of stimuli across participants to eliminate this concern.

Despite these challenges, results from this paper pave the way for future work investigating ecologically valid language and other cognitive tasks using wearable, high-density probes in a larger sample of individuals with stroke-induced aphasia. Additionally, next steps should also consider assessing therapy-related neuroplasticity using fNIRS, given the advantages of using fNIRS to measure brain reorganization associated with language and other cognitive recovery in individuals following neurorehabilitation and limited application in this manner to date.

## Data Availability Statement

The raw data supporting the conclusions of this article will be made available by the authors, without undue reservation.

## Ethics Statement

This study’s procedures involving human participants were reviewed and approved by the Boston University Institutional Review Board. The patients/participants provided their written informed consent to participate in this study.

## Author Contributions

NG acquired and analyzed the fNIRS data, conducted all statistical analyses, generated all figures and tables, and wrote all sections of the manuscript. MY supported the design of the study, including the probe, trained NG to acquire, pre-process, and analyze fNIRS data, and reviewed all aspects of the manuscript. XL optimized the fMRI tasks for use with fNIRS, set up the probe design, built the cap, and completed initial data collection and training of NG. DB provided feedback on the project conceptualization and reviewed the manuscript. SK served as the primary scientific mentor to NG and was involved in project conceptualization, probe design, region of interest selection, management of lesion data, fNIRS data analysis decision making, and manuscript review. All authors contributed to the article and approved the submitted version.

## Conflict of Interest

The authors declare that the research was conducted in the absence of any commercial or financial relationships that could be construed as a potential conflict of interest.

## Publisher’s Note

All claims expressed in this article are solely those of the authors and do not necessarily represent those of their affiliated organizations, or those of the publisher, the editors and the reviewers. Any product that may be evaluated in this article, or claim that may be made by its manufacturer, is not guaranteed or endorsed by the publisher.

## Footnotes

1 At least two wavelengths are used, typically between 650 nm wavelength and 900 nm wavelength. This wide range of wavelengths minimizes crosstalk between chromophores (i.e., molecules that absorb light at a particular frequency) and thus, increases detection of HbO and HbR (i.e., lower wavelength better assessment of HbR, higher wavelength better assessment of HbO).

2 The two groups were not compared statistically due to anatomical and cerebrovascular differences (e.g., greater scalp-to-cortex difference and reduced cerebral blood flow in older individuals) that could influence fNIRS sensitivity to the hemodynamic response (Duncan et al., 1996; Perdue et al., 2012; Karim et al., 2013; Ferreri et al., 2014; Huppert et al., 2017; Csipo et al., 2019).

3 One participant in the stroke group sustained focal traumatic brain injury (i.e., gunshot wound) and consequent middle cerebral artery rupture, left hemisphere stroke, and aphasia.

4 WAB-AQ for one participant was obtained three years prior to the fNIRS measurement. However, this individual presented with chronic aphasia as confirmed by a licensed, certified speech-language pathologist at the time of fNIRS.

5 Semantic feature accuracy and reaction time data was not recorded for seven participants due to a coding error in E-Prime. Arithmetic accuracy and reaction time data was not recorded for two young healthy controls due to a saving error in PsychToolbox-3. Picture naming accuracy could not be scored offline for two older healthy controls and one participant with stroke as the files were not saved in Audacity. Two participants with aphasia did not complete the arithmetic task due to speed. All participants were observed to be completing the task in real time and thus, NIRS data were included in the neural data analyses.

6 MRI could not be acquired at Boston University for one participant due to presence of ferrous material. CT image was obtained from outside hospital, used to draw lesion map, and both were subsequently normalized using Clinical Toolbox for SPM (Rorden et al., 2012).

7
https://www.fil.ion.ucl.ac.uk/spm/software/spm12/

8 More time was allotted to model HRF after the stimulus in the sematic feature verification task than the picture naming and arithmetic tasks because of the difference in task design. Given the potential for cumulative build-up of HRF across the block, there was less concern that additional time after the block was needed to capture the HRF and thus, only 5 s after the stimulus block were modeled for the picture naming and arithmetic tasks. In contrast, given the use of jittered interstimulus intervals, additional time after the stimulus was necessary in the event-related semantic feature task to ensure that HRF was captured for the majority of trials and thus, 7 s (i.e., the length of the average interstimulus interval) after the stimulus item was modeled.
