# Supplementary material for: Investigating Language and Domain-General Processing in Neurotypicals and Individuals With Aphasia — A Functional Near-Infrared Spectroscopy Pilot Study
Source: Front Hum Neurosci. 2021 Sep 17;15:728151. doi: 10.3389/fnhum.2021.728151 (PMC8484538; doi:10.3389/fnhum.2021.728151)
Supplement: Supplementary file 1 [file Table_1.DOCX]

# Supplementary Table 1. fNIRS studies investigating semantic processing, lexical retrieval, and hard vs. easy arithmetic in neurotypicals and individuals with stroke

| Study | | N | | Task | | Regions of interest | | Primary fNIRS data analysis | | Main Findings Relevant to this Study | Primary Limitations |  |
| --- | --- | --- | --- | --- | --- | --- | --- | --- | --- | --- | --- | --- |
| Kennan et al. 2002 | | 6 healthy individuals | | Block design  *- Experimental Condition:* Semantic/Syntactic Decision  *- Control Condition:* Line Decision | | Bilateral inferior frontal areas | | - Laterality index (optodes with positive activation relative to control included, optodes with deactivation excluded) | | - Left hemisphere dominance | - Narrow coverage of the language network  - No short-separation regression or registration to anatomy |  |
| Noguchi et al. 2002 | | 8 healthy individuals | | Event-related design  *- Experimental Tasks:* Syntactic Decision (normal, anomalous)  Semantic Decision (normal, anomalous)  *- Control Task:* Pitch Decision (match, non-match) | | Bilateral frontal temporal, parietal areas | | - Contrasted HbO concentration change in semantic/syntactic decision task conditions with change in pitch task | | - LIFG activation during syntactic, but not semantic processing | - Syntactic stimuli contained semantic anomalies  - No short-separation regression |  |
| Amiri et al. 2014 | | 46 healthy (French-speaking) split into younger and older adults | | Event-related design  *- Experimental Tasks:* Lexical Decision  Words  *- Control Task:* Pseudowords | | Bilateral frontal and temporal areas | | - Continuous wave (TechEn CW6) and time-resolved NIRS  - Calculated both relative and absolute HbO and HbR concentration change  - Investigated effect of condition, age and their interaction  - Accounted for differences in baseline physiology between the groups using measurements from time-resolved NIRS  - Reported significance at p < 0.05 level | | - Older adults showed greater R DLPFC, frontotemporal, occipital temporal and AG activation than controls in the real > pseudoword contrast  - Older adults showed greater RIFG/STG, bilateral DLPFC and LIFG activation in in the pseudoword > real contrast | - Variability in optode positioning affecting group average activation (i.e., smaller group-level activation) |  |
| Cannestra et al .2003 | | - 8 healthy individuals | | Interleaved block design  *- Experimental Tasks:* Finger opposition  Tongue movement  Covert picture naming  *- Control Condition:* Rest | | Left precentral sulcus, IFG, PCG | | - Compared concentration changes in HbO and HbR during task conditions to rest | | - - Left precentral sulcus/IFG active during covert naming - - Change in HbR change (not in HbO) distinguished between tasks/regions | - Narrow coverage of the language network  - Covert naming paradigm (not speech production)  - Compared to rest  - No short-separation regression |  |
| Hull et al. 2009 | | 10 healthy individuals | | Unspecified design  fixation -> line drawing -> rest  - *Experimental Task:* overt line drawing naming  *- Control Condition:* Not specified (included an 8 sec. rest period after each item) | | Bilateral temporal areas  (primary auditory cortices, lateral STG) | | - Compared average HbO concentration change (3-5 second time period) between left and right temporal areas (averaged across channels to create these regions of interest) | | - Left temporal area (HbO increase) upregulated during naming  - Right temporal area downregulated during naming (HbO decrease) | - Narrow coverage of the language network  - No short-separation regression  - Did not compare to active control |  |
| Moriai-Izawa et al. 2012 | | 30 healthy individuals | | Block design  -*Experimental Tasks:* overt line drawing naming  covert line drawing naming  *- Control Condition:* Not specified, task HbO compared to baseline | | Bilateral IFG, PCG, MTG, STG | | - Applied general linear model to the data to obtain HbO and HbR concentration changes for each channel | | - Wider distribution of language areas active during overt (activation in left PCG, IFG, right STG and bilateral MTG) than covert naming (activation in LMTG, LSTG) | - No short-separation regression  - Used canonical HRF versus taking advantage of high temporal resolution in fNIRS and applying basis functions to estimate HRF |  |
| Sakatani et al. 1998 | | 13 healthy individuals,  10 individuals with post-stroke aphasia,  6 individuals without post-stroke aphasia | | Unspecified design  *- Experimental Tasks:* Overt picture naming  Counting 1-50  Talking about what happened yesterday  *- Control Condition:*Not specified (included a 5-10 rest period before task) | | Left PCG | | - Compared concentration changes in HbO, HbR, and HbT from baseline during language tasks using pattern analysis & statistical comparisons of maximum value from pre-activation baseline between participant groups | | - Left prefrontal cortex active during language tasks  - HbR concentration changes distinguished between the three groups, not HbO  - HbR increased during language tasks in post-stroke aphasia group, not the other two groups | - Narrow coverage of the language network  - No short-separation regression  - Incomplete description of fNIRS tasks  - Did not compare to active control  - Predominance of subcortical lesions (10/16) |  |
| Zhang et al. 2017 | | 22 healthy individuals | | Block design  *- Experimental Tasks:* Overt picture naming  *- Control Condition:* Not specified (included a 15-second rest period before task) | | Bilateral frontal, temporal, parietal lobes | | - Voxel-wise analysis of concentration changes in HbO and HbR in object naming versus rest condition using general linear model    - Used principal component analysis spatial filter to remove systemic noise versus short-separation regression | | - HbO results: Deactivation in channels covering left frontal cortex (i.e., rest > naming)  - HbR results: Activation in channels covering LIFGtri, premotor, and supplementary areas (i.e., naming > rest) | | - Did not compare to active control  - Probe appears to cover right hemisphere, but no discussion of results for those regions |
| Artemenko et al. 2018 | | 34 healthy individuals | | Event-related design  - *Experimental Tasks*: Two-digit addition and subtraction  With carry/borrow (hard)  - *Control Condition:* Two-digit addition and subtraction  Without carry/borrow (easy) | | Bilateral frontal, temporal, parietal areas | | - Paired t-tests comparing average HbO and HbR in the hard versus easy conditions of the task  - Split group into high and low math ability | | - High math performers showed  - LIFG activation during hard addition (with carry) & BLIFG and LMFG during hard subtraction (with borrow)  - Low math performers relied on bilateral parietal areas, not frontal | - Used different fNIRS cap sizes, led to “noisier” signal localization in frontal than parietal regions  - No short separation regression |  |
| Artemenko et al. 2019 | | 34 healthy individuals | | Event-related design  - *Experimental Tasks*: Complex multiplication and division  - *Control Condition*: Easy multiplication and division | | Bilateral frontal, temporal, parietal areas | | - Paired t-tests comparing average HbO and HbR in the hard versus easy conditions of the task  - Split group into high and low math ability | | - Greater reliance on frontal activation (LIFG) and lesser reliance on AG, SMG, RMTG during more difficult arithmetic conditions  - High math performers relied on LIFG, LSMG, LSTG for complex math more than low performers | - Response times were of different lengths (participants ended trial themselves or when time ran out after 15 seconds)  - No short separation regression |  |
| *Note.* HbO = oxygenated hemoglobin, HbR = deoxygenated hemoglobin, HbT = total hemoglobin, L = left, R = right, BL = bilateral, IFG = inferior frontal gyrus, AG = angular gyrus, SMG = supramarginal gyrus, MTG = middle temporal gyrus, MFG = middle frontal gyrus, STG = superior temporal gyrus, PCG = precentral gyrus | | | | | | | | | | | |  |
