# Supplementary material for: Investigating Language and Domain-General Processing in Neurotypicals and Individuals With Aphasia — A Functional Near-Infrared Spectroscopy Pilot Study
Source: Front Hum Neurosci. 2021 Sep 17;15:728151. doi: 10.3389/fnhum.2021.728151 (PMC8484538; doi:10.3389/fnhum.2021.728151)
Supplement: Supplementary file 2 [file Table_2.DOCX]

**Supplementary Table 2. Subject N for each ROI**

|  | Task | SF | | | PN | | | Arithmetic | | |
| --- | --- | --- | --- | --- | --- | --- | --- | --- | --- | --- |
| Hemisphere | ROI | YHC | OHC | Stroke | YHC | OHC | Stroke | YHC | OHC | Stroke |
| Left | SFG | 23 | 16 | 5 | 16 | 16 | 4 | 15 | 10 | 3 |
|  | MFG | 24 | 17 | 6 | 17 | 17 | 5 | 16 | 11 | 4 |
|  | IFGtri | 23 | 17 | 5 | 17 | 17 | 4 | 16 | 11 | 3 |
|  | IFGoper | 23 | 16 | 3 | 16 | 16 | 3 | 15 | 10 | 2 |
|  | PCG | 21 | 16 | 3 | 15 | 16 | 3 | 13 | 10 | 2 |
|  | SMG | 20 | 17 | 3 | 15 | 17 | 3 | 14 | 11 | 3 |
|  | MTG | 17 | 16 | 4 | 14 | 16 | 3 | 13 | 10 | 2 |
|  | AG | 17 | 17 | n/a | 12 | 17 | n/a | 11 | 11 | n/a |
| Right | SFG | 24 | 17 | 6 | 17 | 17 | 5 | 16 | 11 | 4 |
|  | MFG | 24 | 16 | 6 | 17 | 17 | 5 | 16 | 11 | 4 |
|  | IFGtri | 24 | 17 | 6 | 17 | 17 | 5 | 16 | 11 | 4 |
|  | IFGoper | 23 | 17 | 6 | 17 | 17 | 5 | 16 | 11 | 4 |
|  | PCG | 21 | 15 | 6 | 15 | 15 | 4 | 14 | 10 | 3 |
|  | SMG | 21 | 17 | 6 | 15 | 17 | 5 | 15 | 11 | 4 |
|  | MTG | 19 | 16 | 6 | 14 | 17 | 5 | 13 | 10 | 4 |
|  | AG | 16 | 16 | 6 | 12 | 17 | 5 | 11 | 11 | 4 |
| *Note.* SF = semantic feature, PN = picture naming, ROI = region of interest, YHC = young healthy controls, OHC = older healthy controls, SFG = superior frontal gyrus, MFG = middle frontal gyrus, IFGtri = inferior frontal gyrus pars triangularis, IFGoper = inferior frontal gyrus pars opercularis, PCG = precentral gyrus, SMG = supramarginal gyrus, MTG = middle temporal gyrus, AG = angular gyrus | | | | | | | | | | |
