# Supplementary material for: Investigating Language and Domain-General Processing in Neurotypicals and Individuals With Aphasia — A Functional Near-Infrared Spectroscopy Pilot Study
Source: Front Hum Neurosci. 2021 Sep 17;15:728151. doi: 10.3389/fnhum.2021.728151 (PMC8484538; doi:10.3389/fnhum.2021.728151)
Supplement: Supplementary file 3 [file Table_3.docx]

**Supplementary Table 3. MNI coordinate, brain labels, and region of interest assignment for long-separation channel**

| Number | MNI Coordinate | "Colin 27" brain atlas label name | Brodmann area | Region of interest assigned |
| --- | --- | --- | --- | --- |
| 1 | -14 57 6 | Frontal_Sup_L | BA10 | LSFG |
| 2 | -9 55 19 | Frontal_Sup_Medial_L | BA10 |  |
| 4 | -23 48 4 | Frontal_Mid_L | BA10 | LMFG |
| 6 | -12 48 19 | Frontal_Sup_Medial_L |  |  |
| 7 | -24 50 21 | Frontal_Sup_L | BA10 |  |
| 10 | -43 50 28 | Frontal_Mid_L |  |  |
| 9 | -30 41 7 | Frontal_Mid_L | BA46 | LIFG pars triangularis |
| 11 | -37 30 21 | Frontal_Inf_Tri_L | BA46 |  |
| 12 | -46 39 10 | Frontal_Inf_Tri_L | BA46 |  |
| 15 | -35 22 22 | Frontal_Inf_Tri_L | BA44 |  |
| 13 | -41 20 12 | Frontal_Inf_Oper_L | BA45 | LIFG pars opercularis |
| 16 | -42 18 25 | Frontal_Inf_Oper_L | BA44 |  |
| 18 | -55 16 28 | Frontal_Inf_Oper_L | BA44 | LPCG |
| 21 | -63 -26 24 | Temporal_Sup_L | BA40 | LSMG |
| 24 | -41 -39 19 | Temporal_Sup_L | BA22 |  |
| 19 | -43 -28 0 | Temporal_Mid_L |  | LMTG |
| 22 | -67 -42 0 | Temporal_Mid_L | BA21 |  |
| 23 | -44 -47 2 | Temporal_Mid_L |  |  |
| 26 | -39 -54 0 | Temporal_Mid_L |  |  |
| 25 | -54 -51 25 | SupraMarginal_L | BA39 | LAG |
| 28 | -46 -56 25 | Angular_L | BA39 |  |
| 29 | 17 56 6 | Frontal_Sup_R |  | RSFG |
| 30 | 18 69 30 | Frontal_Sup_R |  |  |
| 32 | 37 54 4 | Frontal_Mid_R | BA10 | RMFG |
| 34 | 25 60 28 | Frontal_Mid_R |  |  |
| 35 | 31 54 26 | Frontal_Mid_R | BA10 |  |
| 38 | 44 38 24 | Frontal_Mid_R | BA9 |  |
| 37 | 52 44 7 | Frontal_Mid_R | BA46 | RIFG pars triangularis |
| 39 | 49 32 25 | Frontal_Inf_Tri_R | BA9 |  |
| 40 | 46 35 10 | Insula_R | BA46 |  |
| 43 | 50 25 28 | Frontal_Inf_Tri_R | BA9 |  |
| 41 | 64 22 10 | Frontal_Inf_Tri_R | BA44 | RIFG pars opercularis |
| 44 | 65 24 32 | Frontal_Inf_Oper_R |  |  |
| 46 | 66 15 30 | Precentral_R |  | RPCG |
| 49 | 59 -16 24 | Rolandic_Operculum_R | BA40 | RSMG |
| 52 | 48 -30 19 | Rolandic_Operculum_R | BA40 |  |
| 47 | 73 -21 0 | Temporal_Mid_R |  | RMTG |
| 50 | 49 -30 -1 | Temporal_Sup_R | BA22 |  |
| 51 | 73 -46 0 | Temporal_Mid_R |  |  |
| 54 | 58 -51 3 | Temporal_Mid_R | BA37 |  |
| 53 | 62 -45 24 | SupraMarginal_R | BA39 | RAG |
| 56 | 50 -48 25 | Temporal_Sup_R | BA39 |  |
| *Note.* L = Left, R = Right, Sup = Superior, Mid = Middle, Inf = inferior, Tri = triangularis, Oper =opercularis, SFG = superior frontal gyrus, MFG = middle frontal gyrus, IFG = inferior frontal gyrus, PCG = precentral gyrus, SMG = supramarginal gyrus, MTG = middle temporal gyrus, AG = angular gyrus. Channels 3, 8, 17 in the left hemisphere and their corresponding homologues in the right hemisphere (31, 26, 45) were excluded from analyses as their locations did not match across hemispheres and would have resulted imbalanced ROI sizes. BA = Brodmann area | | | | |
