# Supplementary material for: Investigating Language and Domain-General Processing in Neurotypicals and Individuals With Aphasia — A Functional Near-Infrared Spectroscopy Pilot Study
Source: Front Hum Neurosci. 2021 Sep 17;15:728151. doi: 10.3389/fnhum.2021.728151 (PMC8484538; doi:10.3389/fnhum.2021.728151)
Supplement: Supplementary file 4 [file Table_4.DOCX]

**Supplementary Table 4. Number of subjects with all channels in an ROI excluded due to complete damage**

| Task | LSFG | LMFG | IFGtri | IFGoper | PCG | SMG | MTG | AG |
| --- | --- | --- | --- | --- | --- | --- | --- | --- |
| Semantic Feature | 0/0 | 0/2 | 1/5 | 3/4 | 3/3 | 2/4 | 1/3 | 4/4 |
| Picture Naming | 0/0 | 0/2 | 1/4 | 2/3 | 2/2 | 1/3 | 1/2 | 3/3 |
| Arithmetic | 0/0 | 0/2 | 1/3 | 2/3 | 2/2 | 1/3 | 1/2 | 2/2 |
| *Note.* This table shows out of the number of subjects with any damage to the ROI how many subjects had complete lesion damage (i.e., all channels manually excluded). SFG = superior frontal gyrus, MFG = middle frontal gyrus, IFGtri = inferior frontal gyrus pars triangularis, IFGoper = inferior frontal gyrus pars opercularis, PCG = precentral gyrus, SMG = supramarginal gyrus, MTG = middle temporal gyrus, AG = angular gyrus. | | | | | | | | |
