# Supplementary material for: Investigating Language and Domain-General Processing in Neurotypicals and Individuals With Aphasia — A Functional Near-Infrared Spectroscopy Pilot Study
Source: Front Hum Neurosci. 2021 Sep 17;15:728151. doi: 10.3389/fnhum.2021.728151 (PMC8484538; doi:10.3389/fnhum.2021.728151)
Supplement: Supplementary file 5 [file Table_5.DOCX]

| **Supplementary Table 5. Behavioral Data by Group and Task** | | | |
| --- | --- | --- | --- |
|  |  | Accuracy M (SD) | Reaction Time M (SD) |
|  | Semantic Feature Verification | | |
| YHC (n = 17) | Real | 84.09 (6.82) | 1716.96 (289.95) |
|  | Scrambled | 100 (0.00) | 1089.23 (260.05) |
| OHC (n = 17) | Real | 78.87 (9.45) | 2185.4 (310.89) |
|  | Scrambled | 100 (0.00) | 1266.13 (238.32) |
| Stroke (n = 6) | Real | 60.53 (20.05) | 2352.34 (358.88) |
|  | Scrambled | 82.72 (23.36) | 1336.47 (274.66) |
|  | Picture Naming |  |  |
| YHC (n = 17) | Real | 69.83 (11.01) | n/a |
|  | Scrambled | 100 (0.00) |  |
| OHC (n = 15) | Real | 66.11 (13.27) |  |
|  | Scrambled | 97.9 (5.57) |  |
| Stroke (n = 4) | Real | 36.43 (22.73) |  |
|  | Scrambled | 93.57 (10.33) |  |
|  | Arithmetic |  |  |
| YHC (n = 14) | Hard | 95.42 (3.47) | 647.82 (83.83) |
|  | Easy | 98.66 (1.85) | 570.32 (79.28) |
| OHC (n = 11) | Hard | 96.73 (4.69) | 829.01 (227.97) |
|  | Easy | 98.86 (1.35) | 753.21 (196.93) |
| Stroke (n = 4) | Hard | 76.93 (13.51) | 1070.68 (315.41) |
|  | Easy | 91.80 (6.09) | 765.55 (193.73) |
| *Note.* YHC = younger healthy controls, OHC = Older Healthy Controls, M = Mean, SD = standard deviation | | | |
